# Oncogenic osteomalacia: A reversible metabolic bone disorder

**DOI:** 10.4103/0019-5413.67124

**Published:** 2010

**Authors:** Dinesh Kumar Dhanwal

**Affiliations:** Professor of Medicine, Endocrinology Division, Maulana Azad Medical College, New Delhi, India

Oncogenic osteomalacia (OO) or tumor-induced osteomalacia (TIO) is a rare paraneoplastic syndrome and has evolved since its first description as Milkman syndrome in a 15-year-old vitamin D–resistant rickets patient. It is now a well-recognized disorder of defective renal phosphate reabsorption caused by an interplay of various growth factors, chiefly fibroblast growth factor-23 (FGF-23). This syndrome is characterized by severe osteomalacia, normal to low calcium, low phosphorous, raised alkaline phosphatase, and raised levels of FGF-23. In this issue, three interesting cases of OO have been reported in the article ‘Oncogenic osteomalacia – Problems in diagnosis and long-term management’; these cases occurred in association with fibrous dysplasia, polyostotic fibrous dysplasia, and neurofibromatosis. Though the authors have not measured the serum levels of FGF-23 and 1,25-(OH) vitamin D (1α,25(OH)_2_D_3_), there is sufficient circumstantial evidence to support the diagnosis of OO in these three cases. The authors have discussed these cases in detail, highlighting the problems in diagnosis and management.

The key pathogenetic mechanism in OO is dysregulation of phosphorous metabolism. A variety of factors regulate phosphate absorption in the intestine and phosphate reabsorption in the kidney. Parathyroid hormone (PTH) and the vitamin D–endocrine system are the main regulators of phosphate homeostasis. Recently, a number of peptides collectively known as the ‘phosphatonins,’ a term first coined in 1994, have been identified; these include fibroblast growth factor-23 (FGF-23), secreted frizzled-related protein 4 (sFRP-4), fibroblast growth factor-7 (FGF-7), and matrix extracellular phosphoglycoprotein (MEPE).^1^–^3^ These factors have been shown to play a role in the pathogenesis of various hypophosphatemic and hyperphosphatemic disorders, including OO, X-linked hypophosphatemic rickets, and autosomal dominant hypophosphatemic rickets.^4^ FGF-23 is the main peptide to be implicated in the pathogenesis of OO. It is possible that increased circulating levels of FGF-23 cause phosphate wasting through unknown intermediate mechanisms.

The clinical features of OO are reminiscent of that seen in X-linked hypophosphatemia, which serves as the prototype of hypophosphatemic disorders. The clinical features include bony aches and pains, proximal myopathy, pathological fractures, and rachitic deformities in growing children. Onset at a younger age and milder signs and symptoms differentiate X-linked hypophosphatemia from OO. Patients with OO frequently present with fractures and more severe bone pain and muscle weakness than is seen in a hereditary hypophosphatemic disorder. The biochemical hall mark of OO is hypophosphatemia, which results from an excessive loss of phosphate. Other laboratory findings are normocalcemia, elevated alkaline phosphatase, and normal to mildly elevated parathyroid hormone. Low or normal circulating levels of 1α,25(OH)_2_D_3_ are seen despite hypophosphatemia, a major stimulus for 1α,25(OH)_2_D_3_ production. In the Indian context, one should go further and measure 25 OH vitamin D levels also, as vitamin D deficiency is widely prevalent in the Indian population.^5^ With concomitant vitamin D deficiency, one expects aggravation of the symptoms of bone disease in these patients. The partial response seen in one of the patients in the series may well be due to coexisting hypovitaminosis D.

The resolution of these biochemical and bone abnormalities following tumor removal supports the notion of the presence of a circulating factor (phosphatonin) secreted by the tumor. Numerous reports show elevation of FGF-23 in some – but not all – patients with TIO.^2^,^3^ Removal of the tumor is associated with reduction in serum FGF-23 concentrations, and there is a temporal association between the reduction in FGF-23 concentration and the elevation in serum phosphate, decrease in renal phosphate wasting, and increase in 1α,25(OH)_2_D_3_ concentrations.^6^ Elevated levels of FGF-23 may not be diagnostic, as patients with X-lined hypophosphatemia may also have raised levels of this peptide. In the presence of these clinical and laboratory findings one should not leave any stone unturned to localize the tumor. These mesenchymal tumors are often quite small and may not be detected on clinical examination. Most of these are benign and located in the head and neck region and therefore detailed CT and MRI study of the sinuses and jaw should be done. Successful localization of the tumor has been demonstrated using octreotide scintigraphy and PET-CT.^7^,^8^ Despite the use of these modalities, diagnosis may not be possible in a proportion of these patients. Some cases have an occult mesenchymal tumor, which is diagnosed only a few years after the hypophosphatemia is diagnosed. With regard to histological findings, most of these tumors are vascular, with abundant spindle and giant cells, and are reported as hemangiopericytoma.^9^ Treating these patients is a truly gratifying experience as there is dramatic improvement in the osteomalacia after removal of the culprit tumor. The serum phosphate levels, urinary phosphate clearance, and the circulating 1,25-dihydroxyvitamin D return to normal within hours to days. There is complete resolution of the clinical signs and symptoms. In a small group of patients, despite the use of modern imaging modalities, a tumor is not detected, and these patients should be treated with the vitamin D metabolite calcitriol and oral phosphate salt. This treatment may provide some relief to patients till a resectable lesion is identified. Scintigraphy-positive patients awaiting surgery may respond to short-term treatment with parenteral octreotide. The report in the present issue on three cases of OO provide readers with an opportunity to refresh their knowledge about this rare entity.
